# Dynamic-focus transformer for point cloud segmentation

**DOI:** 10.3389/frai.2026.1796099

**Published:** 2026-04-02

**Authors:** Ziwen Wang, Xiaoting Fan, Mei Yu, Jianlu Liu, Shuai Wang, Yonghua Wang, Chuanfu Wu

**Affiliations:** 1 School of Electrical Engineering and Computer Science, University of Ottawa, Ottawa, ON, Canada; 2 Linyi People's Hospital, Linyi, Shandong, China; 3 Shandong Medical College, Linyi, Shandong, China; 4 Shandong Engineering Research Center of Geriatric Health Digital Intelligence Medicine, Linyi, Shandong, China; 5 School of Information Science and Engineering, University of Jinan, Jinan, Shandong, China; 6 Shandong Open Laboratory of Data Innovation Application, Linyi, Shandong, China

**Keywords:** adaptive attention, dynamic-focus, lightweight, point cloud segmentation, transformer

## Abstract

Transformer-based methods have significantly advanced 3D point cloud segmentation by effectively capturing long-range dependencies. However, the global or fixed-window self-attention mechanisms they often employ suffer from computational redundancy and overfitting due to processing excessive, potentially irrelevant key-value pairs for each query. To address this, we propose the Dynamic-Focus Transformer, a novel architecture that introduces a data-dependent adaptive attention mechanism. Through learned soft point masks, we selectively sparsify keys and values to focus on semantically critical regions. Our method enables flexible, input-adaptive receptive fields without the heavy memory overhead associated with per-point offset learning in deformable designs. Furthermore, when integrated into a U-Net-style encoder-decoder, our method attains a highly efficient balance between modeling capability and computational cost. Extensive experiments on S3DIS and ScanNetv2 benchmarks demonstrate that our method achieves state-of-the-art performance with notably improved efficiency, validating its effectiveness for large-scale point cloud understanding.

## 1 Introduction

3D point cloud segmentation is an important technology that serves as a foundation for various applications such as autonomous driving, augmented reality, and robotics. However, 3D point clouds are structurally different from images, preventing the immediate application of standard image-based deep network designs. Numerous deep learning methods (Liu et al., 2023; Li et al., 2025b; Wang et al., 2022; Li et al., 2025a; Fu et al., 2021) on 3D point cloud segmentation have explored solutions to this challenge and achieved promising results. Especially transformer-based methods (Zhao et al., 2021; Fu et al., 2025) have achieved impressive results by taking advantage of transformers to model long-range dependencies.

**Figure 1:**
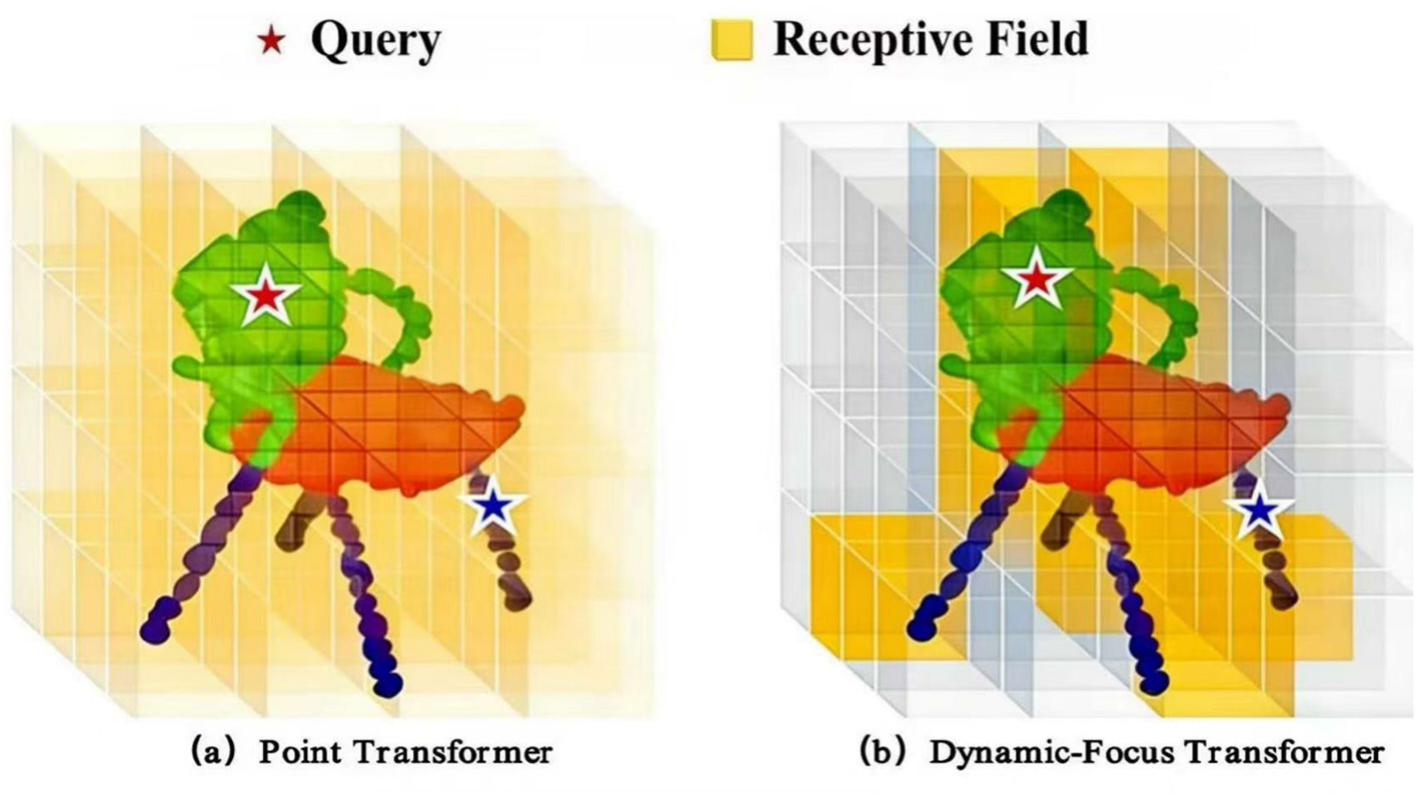
The illustration of our Dynamic-Focus Transformer(DFT). **(a)** Point transformer. **(b)** Dynamic-focus transformer.

Transformer-based point cloud segmentation methods have a larger receptive field and can naturally acquire long-range information through a self-attention mechanism. Point Transformer (Zhao et al., 2021) proposes “vector self-attention” and “subtractive relation” to model the self-attention mechanism, as shown in Figure 1a. However, such global or dense attention over all points is known to incur significant computational redundancy O(*N*^2^) complexity and increases the risk of overfitting when processing large-scale point clouds. Such methods perform non-discriminative attention over the entire spatial region. However, redundant attention in point cloud segmentation leads to computational redundancy and increases the risk of model overfitting. Specifically, an excessive number of keys and values to participate in per query leads to slow convergence and high computational cost, which increases the risk of model overfitting.

Transformer-based point cloud segmentation methods have a larger receptive field and can naturally acquire long-range information through a self-attention mechanism. For instance, Point Transformer (Zhao et al., 2021) employs a global “vector self-attention” mechanism, as shown in Figure 1a. However, such global or dense attention over all points is known to incur significant computational redundancy (O(*N*^2^)complexity) and increases the risk of overfitting when processing large-scale point clouds (Zhao et al., 2021; Liu et al., 2021). To mitigate this, some works in 2D vision have adopted efficient designs. Swin Transformer (Liu et al., 2021) uses fixed local windows to restrict attention, reducing computation but potentially missing long-range context outside the window. Pyramid Vision Transformer (PVT) (Wang et al., 2021) downsamples key/value feature maps, which may discard semantically important information. These approaches highlight a core challenge: balancing modeling capacity with computational efficiency while avoiding overfitting. For 3D point clouds with tens of thousands of points, this trade-off becomes even more critical. Specifically, an excessive number of keys and values for each query leads to high computational cost and slow convergence.

To avoid excessive attention computation, existing works mainly around 2D images utilize well-designed effective attention modules to reduce computation complexity. For example, Swin Transformer (Liu et al., 2021) employs window-based multi-head attention for efficient modeling. Pyramid Vision Transformer (PVT) employs downsampling of key and value feature maps to reduce computational complexity, but this may cause important keys/values to be removed. Ideally, the set of candidate key/values for a given query is flexible and able to adapt to each individual input. In 2D images, Deformable Convolutional Networks (DCN) were proposed to try to address excessive attention computation and achieved impressive results. However, the overhead is huge due to the introduction of deformable offsets in DCN, resulting in high computational complexity. For 3D point clouds with tens of thousands of points, the huge memory consumption and high computational complexity brought by the direct application of DCN are unacceptable.

To address the above issues, we propose a novel Dynamic-Focus Transformer(DFT) for efficient and accurate segmentation. As shown in Figure 1b, unlike fixed-window or dense global attention, our method introduces a data-dependent adaptive attention mechanism that dynamically focuses on semantically important regions through learnable point masks. Specifically, we first fuse coordinate and feature information via a positional representation fusion module, then generate adaptive soft masks for keys and values through a lightweight mask network, which sparsifies candidate tokens per query while preserving essential contextual relationships. This design enables flexible, input-aware receptive fields without the heavy overhead of per-point offset learning as in deformable convolutions, leading to a better efficiency-accuracy trade-off. Integrated into a U-Net-style encoder-decoder, our method effectively models long-range dependencies with reduced computation, accelerates convergence, and mitigates overfitting, making it well-suited for large-scale point cloud understanding. To summarize, our main contributions include the following.

(1) We propose a novel Dynamic-Focus Transformerfor efficient point cloud segmentation, which dynamically learns region-aware masks to select salient keys and values, reducing redundant attention computation while preserving informative context.
(2) We design a lightweight mask network that predicts adaptive attention windows in a data-dependent manner, eliminating the need for handcrafted offset learning as in deformable operators and significantly lowering memory and computational overhead for large-scale point clouds.
(3) Extensive experiments on multiple public benchmarks (e.g., S3DIS, ScanNetv2) demonstrate that our method achieves state-of-the-art performance with notably improved efficiency, offering a favorable trade-off between accuracy and computational cost compared to existing transformer-based point cloud segmentation approaches.

## 2 Related work

### 2.1 Point cloud segmentation

Learning-based methods for point cloud segmentation can be divided into two categories: voxel-based methods and point-based methods. Voxel-based methods convert point clouds into regular representations of 3D voxels. If 3D convolutions are directly applied to 3D voxels, it may incur a large computational cost due to the rapid growth of the number of voxels. The solution is to apply sparse convolutions that take advantage of the sparsity of voxels, which yields good performance. Recently, (Karsli et al., 2024) proposed an improved Octree method for point cloud processing in building extraction, which provides novel insights into the design of efficient voxel structures for point cloud analysis and offers a valuable reference for subsequent comparative research. However, voxel-based methods all suffer from inaccurate position information and loss of geometric details due to voxelization.

Point-based methods directly take the point features and positions of the input point cloud as input, thus keeping the position information and geometric details intact. PointNet leverages permutation-invariant operators such as pooling layers to aggregate features. Many methods such as DGCNN and point website connect point sets into a graph where mutual message passing can take place. Point Transformer (Zhao et al., 2021) aggregates local features using a “vector self-attention” operator, employing full attention for all queries. To improve the accuracy of point cloud analysis, task-oriented point cloud sampling methods have demonstrated prominent advantages by effectively optimizing point cloud sampling strategies. VPI-3DPS (Wang et al., 2026) simulates human visual perception to extract critical point subsets. PointMixSim (Tian et al., 2025) enhances data diversity while preserving key structures. Point-MASNet (Wang et al., 2025) reduces redundancy by learning distinctive local features. These methods are highly effective in optimizing point cloud sampling quality and can work synergistically with our method to further improve the accuracy of point cloud segmentation tasks. Recently, Mamba-based vision models have achieved remarkable progress. The introduction of Mamba into point cloud segmentation (Wang et al., 2024) can reduce computational costs, but its global modeling capability is compromised. Moreover, the region of its context modeling is correlated with the point cloud ordering method, failing to dynamically adjust the modeling range during the modeling process. Point cloud segmentation urgently needs a flexible sparse attention mechanism that adaptively selects important key/value values for a given query. Our work is based on the PointTransformer, but with a fundamental difference: we design an efficient adaptive attention module to overcome the problem of over-attention.

### 2.2 Transformer and deformable CNN

Transformers first achieve success in natural language processing. This inspired the development of transformers for 2D images (Dosovitskiy et al., 2020). Pioneering work Vision Transformer (Dosovitskiy et al., 2020) achieves impressive results on image classification tasks. PVT proposes spatial reduction attention and hierarchical structure to obtain feature pyramids. Swin Transformer (Liu et al., 2021) proposes window-based attention and shifted window operations to achieve improvements in image classification and dense prediction tasks. Inspired by the Swin Transformer, adopts a hierarchical structure and a shift window operation for 3D point clouds. However, the problem of excessive attention computation has not been fully resolved due to the high memory overhead and computational complexity brought by tens of thousands of point cloud data. A solution for flexible selection of key/value sets is urgently needed.

Deformable convolutions (Thomas et al., 2019) aim to attend to flexible spatial locations on input data and are widely used in 2D image-based vision tasks. Recently deformable convolutions have been applied to vision transformers. DPT (Chen et al., 2021) and PS-ViT (Yue et al., 2021) build deformable modules on 2D images to refine visual tokens. Deformable mechanisms are already popular in 2D, but incorporating transformers on point clouds is still underexplored. KPConv (Thomas et al., 2019) attempts to simulate continuous convolution kernels using discrete kernel points on 3D point clouds. But it does not integrate deformable attention into the Transformer-based vision backbone. In contrast, our adaptive attention only needs to learn a set of point masks corresponding to keys and values, so that candidate keys/values are shifted to important regions. Our method can be easily integrated into various Transformer-based vision backbones, making the self-attention module more flexible and efficient.

## 3 Method

### 3.1 Overview

**Figure 2:**
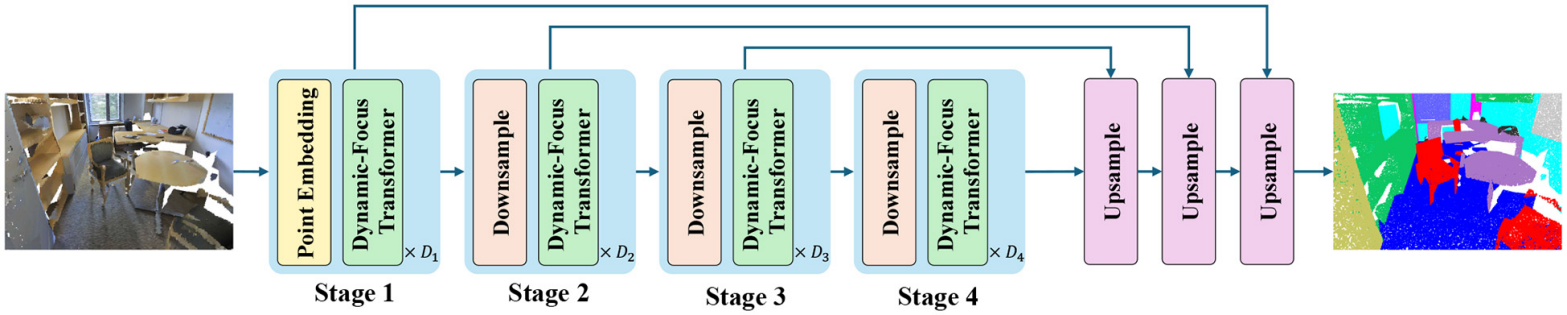
The illustration of our Dynamic-Focus Transformer(DFT) architecture. *D*_1_ to *D*_4_ are the number of stacked Dynamic-Focus Transformer blocks. After the input point clouds are processed through several downsampling layers and Dynamic-Focus Transformerblocks, features are upsampled layer by layer for segmentation.

Given a point cloud $P={\left\{\left({\text{p}}_{n},{\text{c}}_{n}\right)\right\}}_{n=1}^{N}$ with *N* points, we use both *xyz* coordinates **p** ∈ ℝ^*N*×3^ and *rgb* colors **c** ∈ ℝ^*N*×3^ as input to our model. Our framework is point-based and employs an encoder-decoder structure. An overview of our method is shown in Figure 2. The encoder consists of multiple stages. Except for the first stage which locally aggregates the raw input using a layer of point embedding module. The rest of the stages are connected by downsampling layers and our Dynamic-Focus Transformer layers, which are implemented by the adaptive attention mechanism proposed in this paper. For the decoder, the features of the encoder's Dynamic-Focus Transformer layers are upsampled layer by layer in a manner similar to U-Net (Ronneberger et al., 2015).

### 3.2 Vanilla transformer block

In this section, we first revisit the attention mechanism in recent Point Transformers. Vanilla global self-attention results in massive memory consumption *O*(*N*^2^), where N is the number of input points. Recent window-based self-attention achieves a significant reduction in memory complexity by partitioning 3D space into non-overlapping cubic windows. In window-based self-attention, each query point does not need to consider all points, but neighbor points in the same window. We denote *k*_*t*_ as the number of points within the *t*-th window. Given points $\text{x}\in {\mathbb{R}}^{k_{t}\times \left(N_{h}\times N_{d}\right)}$ as input, where *N*_*h*_ is the number of heads and *N*_*d*_ is the dimension of each head, a window-based multi-head self-attention (WMHA) block is represented in *t*-window as:


$$\begin{array}{l} \text{q}=\varphi_{q}\left(\text{x}\right),\text{k}=\varphi_{k}\left(\text{x}\right),\text{v}=\varphi_{v}\left(\text{x}\right) \end{array}$$ (1)

$$\begin{array}{l} {\text{attn}}_{h}=\sigma \left({\text{q}}_{h}\cdot {\text{k}}_{h}\right) \end{array}$$ (2)

$$\begin{array}{l} {\text{y}}_{h}=\psi \left(\overset{{\text{k}}_{t}}{\underset{j=1}{\sum }}{\text{attn}}_{j,h}\times {\text{v}}_{h}\right) \end{array}$$ (3)

where $\text{q},\text{k},\text{v}\in {\mathbb{R}}^{k_{t}\times \left(N_{h}\times N_{d}\right)}$ denote query, key, and value embeddings respectively. φ_*q*_, φ_*k*_, φ_*v*_ and ψ are linear projections. σ(·) denotes the softmax function, and **y** is the output feature.

Typically, to build a point transformer block, an MLP block with a GELU activation and two linear projections is used to provide the nonlinearity. The *l*-th point transformer block is denoted as:


$$\begin{array}{l} {\text{y}}^{l}=WMHA\left(LN\left({\text{y}}^{l-1}\right)\right)+{\text{y}}^{l-1} \end{array}$$ (4)

$$\begin{array}{l} {\text{z}}^{l}=MLP(LN({\text{y}}^{l})+{\text{y}}^{l} \end{array}$$ (5)

where *z*^*l*^ is the output of the *l*-th point transformer block, LN is the layer normalization.

### 3.3 Adaptive attention

Existing 3D point cloud transformers, especially Point Transformer (Zhao et al., 2021) and Fast Point Transformer (Park et al., 2022) try to use transformer to model all receptive fields, which brings redundant attention computation. Similar to Vision Mamba (Zhu et al., 2024) that extends Mamba to 2D vision tasks, Mamba-based point cloud segmentation methods (Wang et al., 2024; Liang et al., 2024) leverage its sequential state-space model to achieve higher computational efficiency compared with transformer-based methods. However, due to Mamba's inherent reliance on ordered point cloud sequences and fixed state transition rules, its capability to capture global contextual dependencies is significantly constrained. This limitation restricts its performance in modeling large-scale point cloud scenes or objects with complex structural relationships. Therefore, a data-dependent sparse attention is urgently needed to flexibly model point cloud-related representations. To address this issue, we propose an adaptive attention mechanism. A similar sparse attention mechanism in 2D is the deformable mechanism proposed in DCN (Dai et al., 2017). Since each element in the deformable mechanism learns its offset separately, if we directly apply the same mechanism in the point cloud attention module, the space complexity will rise sharply. We opt for a simpler solution that is more suitable for point cloud data, providing adaptive keys and values for each query to achieve an efficient trade-off.

**Figure 3:**
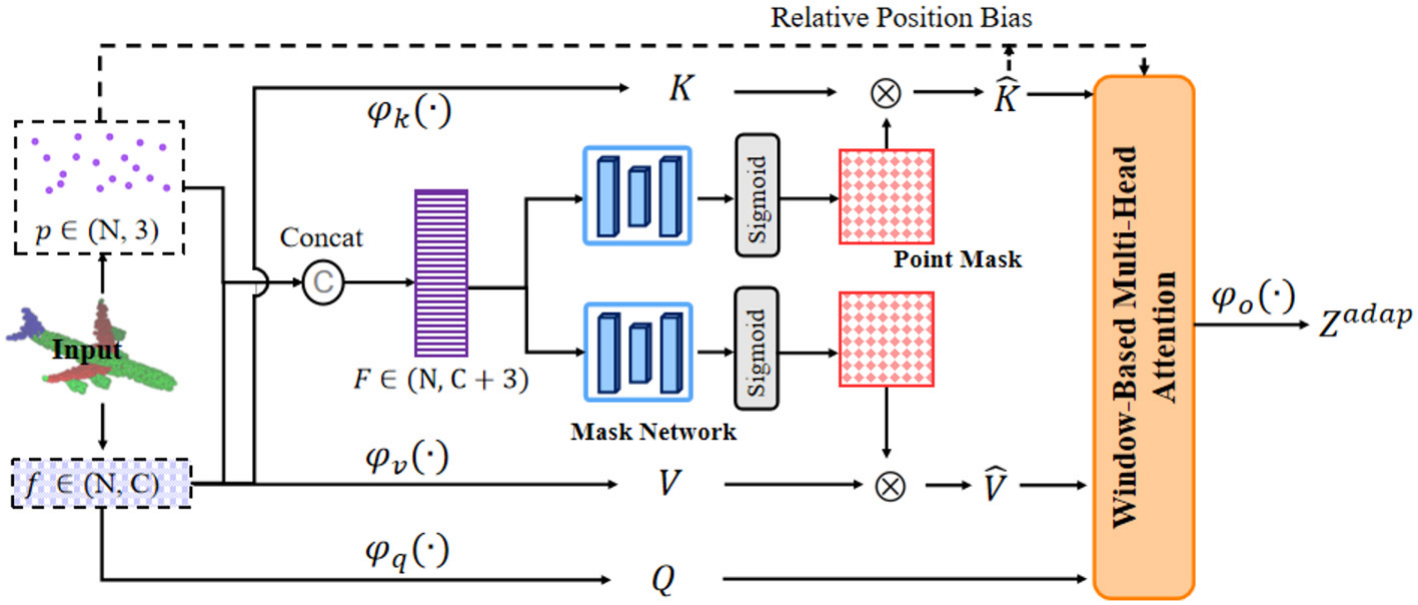
The illustration of our adaptive attention mechanism. The coordinate information and feature information of the input point clouds are first processed by the positional representation fusion mechanism to generate aggregate features and relative position encoding. The aggregated features are then fed into the mask network to generate point masks. Adaptive keys and adaptive values are obtained according to the learnable point mask.

Specifically, we propose adaptive attention to selectively model the relationship between tokens guided by important regions in point clouds. These important regions are identified by a learnable point mask. As shown in Figure 3, we first fuse the positional information representation via the proposed Positional Representation Fusion Mechanism to obtain aggregated feature and relative position encoding. The aggregated feature is then fed into the mask network to generate point masks. Finally, point masks are used to get the adaptive key and value. Adaptive attention can flexibly model point cloud-related representations, which will be discussed in the following sections.

#### 3.3.1 Positional representation fusion mechanism

Let $P={\left\{\left({\text{p}}_{n},{\text{f}}_{n}\right)\right\}}_{n=1}^{N}$ be the input points, where *N* is the total number of point clouds, **p** ∈ ℝ^*N*×3^ is the point coordinate and **f** ∈ ℝ^*N*×*C*^ is the corresponding input point features. We divide the input point clouds P into non-overlapping cubic windows, and the points of the *t*-th window is denoted as (**p**_*t*_, **f**_*t*_). We obtain the aggregated feature **F**_*t*_ fusing positional representations in the *t*-th window by simply concatenating **p**_*t*_ and **f**_*t*_ together:


$$\begin{array}{l} {\text{F}}_{t}=Concat\left({\text{p}}_{t},\;{\text{f}}_{t}\right) \end{array}$$ (6)

To obtain relative positional encodings, we define three learnable lookup tables **T**_1_, **T**_2_, **T**_3_ corresponding to the x, y, and z axes, respectively. Based on Zhao et al. (2021), we look up the table to retrieve the corresponding embeddings, and sum them, resulting in the positional encoding δ:


$$\begin{array}{l} \delta =\overset{3}{\underset{k=1}{\sum }}{\text{T}}_{k}\left[\frac{1}{s_{quant}}\left({\text{p}}_{i,k}-{\text{p}}_{j,k}+s_{win}\right)\right] \end{array}$$ (7)

where $\text{T}\left[idx\right]\in {\mathbb{R}}^{N_{h}\times N_{d}}$ represents the *idx*-th entry of table *T*, *s*_*win*_ is the window size, and *s*_*quant*_ is the quantization size. Since the tables for query, key, and value are not shared, we denote the positional encodings corresponding to query, key, and value by δ_*q*_, δ_*k*_, and δ_*v*_, respectively.

#### 3.3.2 Point mask generation

For the aggregated feature **F**, we feed it into two mask networks with the same structure respectively. The purpose of the mask network is to predict the point mask of the key ${\text{m}}_{k}\in {\mathbb{R}}^{N\times 1}$ and the point mask of the value ${\text{m}}_{v}\in {\mathbb{R}}^{N\times 1}$ separately. To achieve efficient computing, the mask network is composed of three linear transformations and a sigmoid activation function to make the output between 0 and 1. The **m**_*k*_ and **m**_*v*_ are calculated by:


$$\begin{array}{l} {\text{m}}_{k}=\theta_{mask}^{k}\left(\text{F}\right),\;{\text{m}}_{v}=\theta_{mask}^{v}\left(\text{F}\right) \end{array}$$ (8)

For each query, we get the corresponding point mask **m**_*k*_ and **m**_*v*_. For **m**_*k*_ and **m**_*v*_, we dot product them with vanilla key **k** and vanilla value **v** respectively to get the adaptive key $\hat{\text{k}}$ and adaptive value $\hat{\text{v}}$:


$$\begin{array}{l} \hat{\text{k}}={\text{m}}_{k}\cdot \text{k},\;\hat{\text{v}}={\text{m}}_{v}\cdot \text{v} \end{array}$$ (9)

#### 3.3.3 Adaptive attention module

Combining the above modules, we formalize the adaptive attention module as follows. Given an input points $P={\left\{\left({\text{p}}_{n},{\text{f}}_{n}\right)\right\}}_{n=1}^{N}$, we get the aggregated feature **F** and relative positional encodings via Positional Representation Fusion Mechanism, and get **m**_*k*_ and **m**_*v*_ via Point Mask Generation. We first get the query, adaptive key and adaptive value with the following formula:


$$\begin{array}{l} \text{q}=\varphi_{q}\left(\text{f}\right),\hat{\text{k}}={\text{m}}_{k}\cdot \varphi_{k}\left(\text{f}\right),\hat{\text{v}}={\text{m}}_{v}\cdot \varphi_{v}\left(\text{f}\right) \end{array}$$ (10)

where ${\text{m}}_{k},{\text{m}}_{v}\in {\mathbb{R}}^{N\times 1}$, $\hat{\text{k}}$ is the adaptive key for query **q**, $\hat{\text{v}}$ is the adaptive value for query **q**. We define positional bias as the sum of dot products of positional encodings with query and adaptive key features, respectively. The process is formulated as:


$$\begin{array}{l} \text{bias}=\text{q}\cdot \delta_{q}+\hat{\text{k}}\cdot \delta_{k} \end{array}$$ (11)

We perform window-based multi-head attention on **q**, **k**, **v** and employ a relative positional bias **bias**. We define *M* as the total number of heads. For points in *t*-window, the output of *m*-th attention head is formulated as:


$$\begin{array}{l} {\text{attn}}_{m}^{adap}=\sigma \left({\text{q}}_{m}\cdot {\hat{\text{k}}}_{m}+{\text{bias}}_{m}\right) \end{array}$$ (12)

$$\begin{array}{l} {\text{y}}_{m}^{adap}=\overset{k_{t}}{\underset{j=1}{\sum }}attn_{j,m}^{adap}\times \left({\text{v}}_{m}+\delta_{v}\right) \end{array}$$ (13)

$$\begin{array}{l} z_{m}^{adap}=\varphi_{o}\left(y_{m}^{adap}\right) \end{array}$$ (14)

where *z*^*adap*^ is the output of our Dynamic-Focus Transformerblock, φ_*o*_ is the linear projection of the output.

#### 3.3.4 Computational complexity

For the input points of size *N*×*C*, the complexity of the vanilla multi-head attention is *O*(4*NC*^2^+2*N*^2^*C*), The computational cost of Adaptive Multi-Head Attention (AMHA) is similar to that of Swin Transformer. In fact, since some weights in the point mask are 0, the calculation cost of AMHA's multi-head attention is even lower than that of vanilla multi-head attention, and the only additional overhead comes from the sub-network used to generate the point mask. The complexity of the entire module can be summarized as:


$$\begin{array}{l} \text{Ω}\left(AMHA\right)=2KNC+2KC^{2}+2NC^{2}+2\text{Ω}\left(\theta_{mask}\right) \end{array}$$ (15)

$$\begin{array}{l} \text{Ω}\left(\theta_{mask}\right)=\left(C+3\right)\left(C/2\right)N+3\left(C/2\right)N+3N\\ \;=\left(\left(C+6\right)\left(C/2\right)+3\right)N \end{array}$$ (16)

where *K* is the number of effective points whose weight is not 0. Considering that the computational cost of the mask network has linear complexity w.r.t. the size of the channel, we set *C* to a small value of 48. Therefore, applying our Adaptive Attention mechanism is efficient.

### 3.4 Network architecture

Based on the above modules, we develop an Dynamic-Focus Transformerfor point cloud segmentation. Regarding network architecture, our Dynamic-Focus Transformerhas a similar encoder-decoder structure to Zhao et al. (2021). For the S3DIS dataset, we build four stages with block depth [2, 2, 6, 2]. While for the ScanNetv2 dataset, due to its larger number of points, we construct five stages with block depths [3, 3, 9, 3, 3]. Taking the backbone consisting of 4 stages as an example, as shown in Figure 3, between two consecutive stages, there is a downsampling layer to downsample the point cloud, halving the number of points. Therefore, the base of the point set generated in each stage is [*N, N*/4, *N*/4^2^, *N*/4^3^, *N*/4^4^], where *N* is the number of input points. The network architecture is shown in Figure 3, and the input point clouds contain *xyz* coordinates and point features (*rgb* colors). For the S3DIS dataset, we set the number of channels to [48, 96, 192, 384] and the number of heads to [3, 6, 12, 24]. For the ScanNetv2 dataset, we set the number of channels to [48, 96, 192, 384, 384] and the number of heads to [3, 6, 12, 24, 24].

## 4 Experiments

### 4.1 Datasets and evaluation metric

**S3DIS**. The S3DIS (Armeni et al., 2016) dataset has 13 semantic categories and consists of 271 rooms in 6 regions. Following common practice (Zhao et al., 2021), we test on Area 5 and utilize other areas for training. Furthermore, we use the common overall pointwise accuracy (OA), mean of classwise accuracy (mAcc), and mean classwise intersection over union (mIoU) as evaluation metrics.

**ScanNetv2**. The ScanNetv2 dataset contains 1613 indoor RGB-D scenes, of which 1201 scenes are used for training, 312 scenes are used for validation and 100 scenes are used for testing. And ScanNetv2 is annotated with semantic labels within 20 categories. We evaluate our method on ScanNetv2. In addition, We adopt mIoU as the evaluation metric.

### 4.2 Implementation details

We implement the proposed Dynamic-Focus Transformerin PyTorch and use the AdamW optimizer with an initial learning rate of 0.006. All experiments are performed on 4 RTX A6000 GPUs. For semantic segmentation on S3DIS, we train for 76,500 iterations with weight decay and momentum set to 0.01 and 0.9, respectively. The batch size is set to 4. Following previous work, the original input points are sampled with a grid size of 0.04m. Furthermore, We adopt a series of data augmentation strategies, i.e., z-axis rotation, jitter and scale. to enhance the generalization capability of the model. For semantic segmentation on ScanNetv2, we train for 600 epochs and set the batch size to 8. Weight decay and momentum are set to 0.01 and 0.9, respectively. We sample the input points with a grid size of 0.02m. Except for random jitter, the data augmentation strategy is the same as the setting on S3DIS.

### 4.3 Results

#### 4.3.1 Performance comparison on S3DIS

The results of S3DIS dataset is shown in Table 1. Compared to recent state-of-the-art semantic segmentation methods, our Dynamic-Focus Transformer(DFT) achieves best performance on both challenging datasets. On S3DIS, DFT attains OA/mAcc/mIoU of 91.5%/78.4%/72.5%, outperforming all previous works on every metric. Our method even outperforms the 2.1% mIoU of point transformers.

**Table 1:** Segmentation mIoU (%) on the S3DIS Area5.

Method	Input	OA	mAcc	mIoU
PointNet; Qi et al. (2017a)	point	-	49.0	41.1
SegCloud; Tchapmi et al. (2017)	point	-	57.4	48.9
TangentConv; Tatarchenko et al. (2018)	point	-	62.2	52.6
PointCNN; Li et al. (2018)	point	85.9	63.9	57.3
PointWeb; Zhao et al. (2019)	point	87.0	66.6	60.3
HPEIN; Jiang et al. (2019)	point	87.2	68.3	61.9
GACNet; Wang et al. (2019)	point	87.8	-	62.9
PAT; Yang et al. (2019)	point	-	70.8	60.1
ParamConv; Wang et al. (2018)	point	-	67.0	58.3
SPGraph; Landrieu and Simonovsky (2018)	point	86.4	66.5	58.0
SegGCN; Lei et al. (2020)	point	88.2	70.4	63.6
MinkowskiNet; Choy et al. (2019)	voxel	-	71.7	65.4
PAConv; Xu et al. (2021)	point	-	-	66.6
KPConv; Thomas et al. (2019)	point	-	72.8	67.1
SerializedPointMamba; Wang et al. (2024)	point	-	-	70.6
PointTransformer; Zhao et al. (2021)	point	90.8	76.5	70.4
DFT	point	**91.5**	**78.4**	**72.5**

The bold values represent the best results.

#### 4.3.2 Performance comparison on ScanNetv2

The results of S3DIS dataset is shown in Table 2. The test mIoU of our method is 73.7%. It outperforms others models including voxel-based models such as MinkowskiNet (Choy et al., 2019), and point-based models such as PointTransformer (Zhao et al., 2021). On the validation set, we also obtain higher results than other state-of-the-art methods, proving the effectiveness of our adaptive attention.

**Table 2:** Segmentation mIoU (%) on the ScanNetv2.

Method	Input	Val mIoU	Test mIoU
PointNet++; Qi et al. (2017b)	point	53.5	55.7
3DMV; Dai and Nießner (2018)	point	-	48.4
PanopticFusion; Narita et al. (2019)	point	-	52.9
PointCNN; Li et al. (2018)	point	-	45.8
PointConv; Wu et al. (2019)	point	61.0	66.6
JointPointBased; Chiang et al. (2019)	point	69.2	63.4
PointASNL; Yan et al. (2020)	point	63.5	66.6
SegGCN; Lei et al. (2020)	point	-	58.9
RandLA-Net; Hu et al. (2020a)	point	-	64.5
KPConv; Thomas et al. (2019)	point	69.2	68.6
JSENet; Hu et al. (2020b)	point	-	69.9
FusionNet; Zhang et al. (2020)	point	-	68.8
PointTransformer; Zhao et al. (2021)	point	70.6	-
SparseConvNet; Graham et al. (2018)	voxel	69.3	72.5
MinkowskiNet; Choy et al. (2019)	voxel	72.2	73.6
DFT	point	**73.8**	**74.0**

The bold values represent the best results.

#### 4.3.3 Performance comparison on computational efficiency

The Table 3 presents a side-by-side comparison of your proposed Dynamic-Focus Transformer (DFT) method against two key baseline methods, PointTransformer and KPConv, across the aforementioned metrics. This aims to visually demonstrate DFT's computational efficiency advantages while maintaining accuracy.

**Table 3:** Comparison of computational efficiency.

Method	GFLOPs	Parameters (M)	Inference Time (ms)
PointTransformer; Zhao et al. (2021)	5.6	9.5	285
KPConv; Thomas et al. (2019)	14.8	14.8	320
DFT (Ours)	**3.9**	**7.2**	**210**

Efficiency metrics are measured on a single RTX A6000 GPU with a batch size of 1 and 40,960 input points, using the ptflops toolkit for FLOPs calculation. The bold values represent the best results.

### 4.4 Qualitative results and visualization

**Figure 4:**
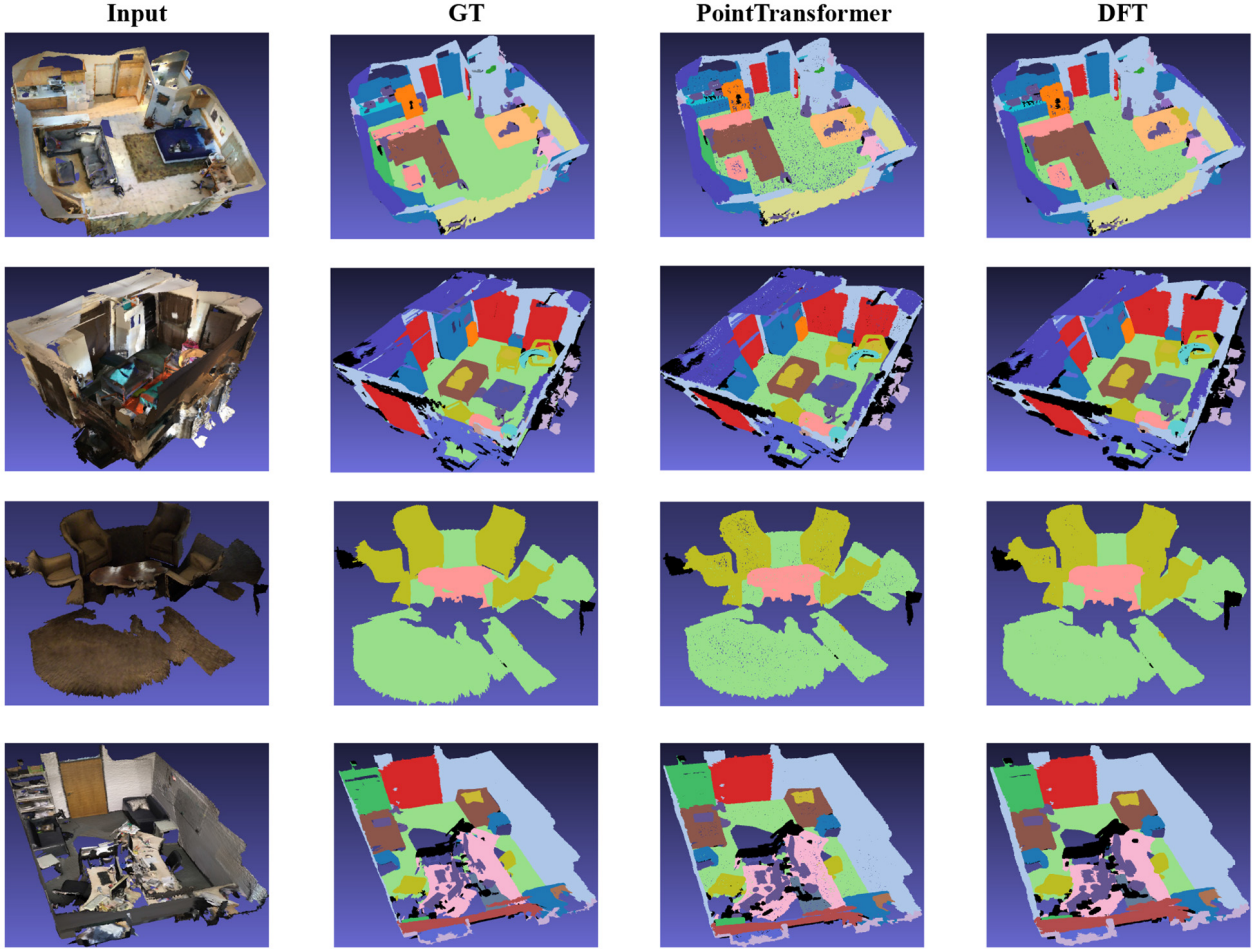
Qualitative comparison of semantic segmentation results on ScanNetv2: Input image, ground truth, DFT, and PointTransformer.

Figure 4 presents a qualitative comparison of semantic segmentation results from the ScanNetv2 dataset. The rows show different challenging scenes from the dataset, and for each scene, four columns are displayed:

- **Input**: The raw input image, which represents the 3D point cloud data captured from the scene.
- **GT (Ground Truth)**: The ground truth segmentation, where each object or region in the scene is manually annotated with semantic labels, serving as the reference for evaluation.
- **PointTransformer**: The segmentation result produced by the PointFormer model, which is another state-of-the-art method for point cloud segmentation.
- **DFT (Dynamic-Focus Transformer)**: The segmentation result produced by the proposed Dynamic-Focus Transformer (DFT) method. This method uses an adaptive attention mechanism to focus on semantically important regions while maintaining computational efficiency.

The DFT method is compared visually to PointTransformer to demonstrate its ability to produce more accurate and coherent segmentations, especially in fine-structure and boundary regions. In the scenes displayed, DFT appears to handle complex and fine-grained regions better than PointTransformer, particularly in regions with intricate boundaries and varying object structures, leading to more accurate segmentation. This reinforces the method's ability to reduce overfitting and improve efficiency while maintaining segmentation quality.

### 4.5 Ablation studies

In this section, we conduct extensive ablation experiments to examine specific decisions in DFT design.

#### 4.5.1 Adaptive attention transformer

To verify the effectiveness of each component of DFT, we report the ablation experiments on the S3DIS and ScanNetv2 datasets in Table 4. After incorporating the Positional Representation Fusion Mechanism (PRFM), the model's mIoU on S3DIS increased from 68.1% to 69.5% (a gain of 1.4%), and on ScanNetv2 from 69.5% to 70.8% (a gain of 1.3%). The subsequent integration of the point masks **m**_*k*_ and **m**_*v*_, which adaptively guide the selection of candidate keys and values, further enhanced the flexibility and efficiency of our attention mechanism. This led to an additional improvement, raising the mIoU to 70.8% on S3DIS and 72.1% on ScanNetv2. Finally, the inclusion of the positional bias (Pos. Bias) is indispensable, completing our DFT and yielding the final performance of 72.5% mIoU on S3DIS and 74.0% on ScanNetv2. The results demonstrate that each proposed component contributes positively to the overall performance.

**Table 4:** Ablation studies on the components of the Adaptive Attention Transformer.

PRFM	m_k	m_v	Pos. Bias	Method	S3DIS (mIoU%)	ScanNetv2 (mIoU%)
				Baseline	68.1	69.5
✓					69.5	70.8
✓	✓				70.8	72.1
✓	✓	✓		DFT (w/o Pos. Bias)	71.6	73.0
✓	✓	✓	✓	DFT (Ours)	**72.5**	**74.0**

PRFM, Positional Representation Fusion Mechanism. The bold values represent the best results.

#### 4.5.2 Positional representation fusion mechanism

We conducted an ablation study on the Positional Representation Fusion Mechanism in DFT. The results are shown in Table 5. It can be seen that without fused positional representations, the performance drops significantly to an mIoU of 40. *w*/*oaggregatedfeature* means that there is no aggregated feature and point features are used as the input of the mask network. The results in Table 1 show that no aggregated feature or no positional encodings will significantly affect the model performance, indicating that the fusion of positional representations in self-attention is extremely important for point cloud segmentation.

**Table 5:** Ablation study: Positional Representation Fusion Mechanism.

Pos. Representation	OA	mAcc	mIoU
none	-	59.0	65.1
w/o aggregated feature	-	63.7	68.2
w/o positional encodings	-	69.1	71.8
DFT	**91.5**	**78.4**	**72.5**

The bold values represent the best results.

#### 4.5.3 Adaptive attention module

Finally, we explore the types of self-attention used in Dynamic-Focus Transformer layers and variants of each component in the adaptive attention module. The reslut as shown in Table 6. Based on the result, the proposed Adaptive Attention significantly outperforms alternative attention variants. While Deformable Attention incurs high computational overhead and achieves suboptimal results 74.6% mACC and 70.3% mIoU, our method attains superior performance 78.4% mACC and 72.5% mIoU. The results validate that Adaptive Attention more effectively and efficiently captures salient point cloud features.

**Table 6:** Ablation study: Adaptive Attention Module.

Attention variants	OA	mAcc	mIoU
Deformable attention	-	74.6	70.3
Scalar attention	-	71.5	65.2
MLP mask network	-	69.2	63.1
DFT	**91.5**	**78.4**	**72.5**

The bold values represent the best results.

#### 4.5.4 Hyperparameter analysis

To validate our design choices and investigate the model's robustness in Table 7, we conducted a sensitivity analysis on key hyperparameters of the Dynamic-Focus Transformer, with the results summarized in the provided table. All experiments were performed on Area 5 of the S3DIS dataset, using the performance of our default configuration (learned Sigmoid masks, channel number C = 48, window size 8 × 8 × 8), which achieves 72.5% mIoU, as the baseline.

**Table 7:** Sensitivity analysis of key hyperparameters in our Dynamic-Focus Transformer (DFT).

Configuration variant	mIoU
A. Variants of the Mask Mechanism	
1. Fixed Threshold (0.5)	71.6 (↓0.9)
2. Learned Sigmoid Masks (*default*)	**72.5**
3. MLP Mask Network (*2 layers*)	71.9 (↓0.6)
B. Mask Network Channels (C)	
C = 24	71.8 (↓0.7)
C = 48 (*default*)	**72.5**
C = 96	72.3 (↓0.2)
C. Window Size	
4 × 4 × 4	71.1 (↓1.4)
8 × 8 × 8 (*default*)	**72.5**
12 × 12 × 12	71.9 (↓0.6)

The bold values represent the best results.

## 5 Conclusion

In this paper, we presents the Adaptive Attention Transformer, a novel and efficient architecture for 3D point cloud segmentation. By proposing a lightweight mask network that dynamically generates point-wise masks, our method selectively attends to semantically salient regions, effectively addressing the issue of redundant attention computation. This data-dependent sparse attention mechanism enables flexible, input-aware receptive fields without incurring the memory and computational overhead associated with per-point offset learning. Extensive experiments on S3DIS and ScanNetv2 datasets demonstrate that our method achieves state-of-the-art performance in semantic segmentation, while offering a good balance between accuracy and efficiency. Our work validates the importance of adaptive sparse attention for large-scale point cloud understanding and provides a practical foundation for future research in efficient 3D vision architectures.

## Data Availability

Publicly available datasets were analyzed in this study. This data can be found here: https://cvg-data.inf.ethz.ch/s3dis/.
